# Moisture Detection in Tree Trunks in Semiarid Lands Using Low-Cost Non-Invasive Capacitive Sensors with Statistical Based Anomaly Detection Approach

**DOI:** 10.3390/s23042100

**Published:** 2023-02-13

**Authors:** Ashraf Ali, Ahmad Ali, Hussein Abaluof, Wafaa N. Al-Sharu, Omar A. Saraereh, Andrew Ware

**Affiliations:** 1 Electrical Engineering Department, Faculty of Engineering, The Hashemite University, Zarqa 13133, Jordan; 2 Computer Systems Institute, 529 Main Street, Charlestown, MA 02129, USA; 3 Huawei Technologies, Amman 11183, Jordan; 4 Faculty of Computing, Engineering and Sciences, University of South Wales, Pontypridd CF37 1DL, UK

**Keywords:** sensors, capacitive sensor, wood trunks, oscillator, dielectric material, smart agriculture, LoRa, anomaly detection, eigenvectors, eigenvalues, covariance matrix

## Abstract

This paper focuses on building a non-invasive, low-cost sensor that can be fitted over tree trunks growing in a semiarid land environment. It also proposes a new definition that characterizes tree trunks’ water retention capabilities mathematically. The designed sensor measures the variations in capacitance across its probes. It uses amplification and filter stages to smooth the readings, requires little power, and is operational over a 100 kHz frequency. The sensor sends data via a Long Range (LoRa) transceiver through a gateway to a processing unit. Field experiments showed that the system provides accurate readings of the moisture content. As the sensors are non-invasive, they can be fitted to branches and trunks of various sizes without altering the structure of the wood tissue. Results show that the moisture content in tree trunks increases exponentially with respect to the measured capacitance and reflects the distinct differences between different tree types. Data of known healthy trees and unhealthy trees and defective sensor readings have been collected and analysed statistically to show how anomalies in sensor reading baseds on eigenvectors and eigenvalues of the fitted curve coefficient matrix can be detected.

## 1. Introduction

When measuring humidity in the agriculture sector, soil moisture sensors are commonly used to detect moisture in the soil at different depths. There are four types of soil moisture sensors: resistance blocks, neutron thermalization, capacitance sensing (frequency domain sensing), and travel time sensing (time-domain transmission modes and time-domain reflectometry). Of the four, capacitance sensing (frequency domain sensing) is the most used and measures the variation in capacitance between two probes. The soil moisture sensor is based on fringing capacitance and the increase in the dielectric constant of a soil–water mixture with water content.

**Figure 1:**
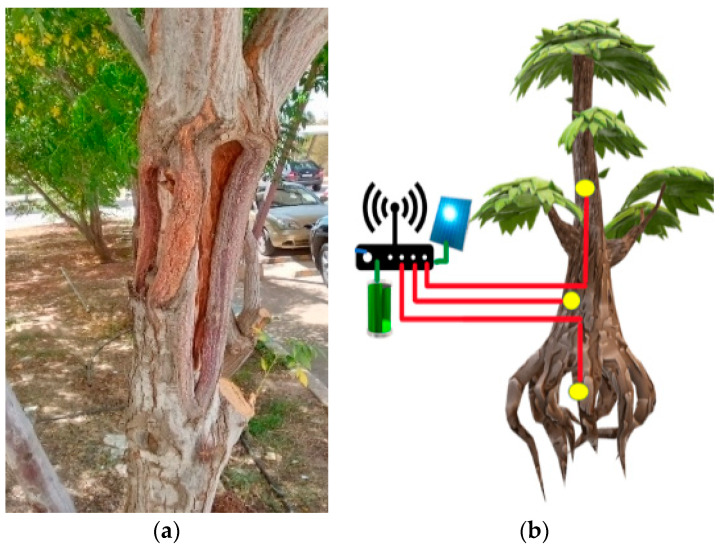
(**a**) External deformations in tree trunks. (**b**) Setup of sensors, microcontroller, and communication interface.

The frequency shifts of an RC oscillator connected to the capacitance sensor electrode are then measured by a microcontroller. However, soil sensors cannot detect moisture in tree trunks and branches. Despite the direct correlation between the moisture content of the soil and tree trunks’ moisture, there are situations in which there could be variations between the two parameters. Examples include severe tree trunk damage to the chinaberry tree, whose scientific name is *Melia Azedarach*, as shown in Figure 1a, insect damage to tree trunk tissue, a disease that prevents the tree from extracting water normally, or a fungus that affects the tree’s roots. All such factors cause significant variation between soil and trunk moisture content. This may cause the soil sensor to mistakenly indicate that the tree is well hydrated. Hence, a sensor capable of measuring the moisture inside the tree trunk is thought to be more accurate and useful than measuring the water in the soil.

Time Domain Reflectometers (TDRs) have been used intensively to measure tree trunks’ moisture. The sensor probes are implanted in the trunk, and a coaxial cable guides the signal into the reading panel. The cost of such sensors is high due to the high-frequency oscillators built inside them and the time delay detection circuitry that needs to detect delays in Pico seconds. The cost and the invasive probes are two of the downsides of such sensors. Moreover, trees located at a distance from the data processing unit mean that a scalable solution with a connectivity interface that can transmit the data in real-time is a significant challenge.

A wireless technology to transfer the data to the processing unit is thus a better option. Using LoRaWan to facilitate this transfer has proven to be an effective way to achieve this. The connectivity can be achieved via connecting all the sensors directly with a gateway or connecting the nodes in an ad-hoc manner until the message is transferred to the gateway to deliver these readings for a very long range. Figure 1b shows the general connectivity of such a sensor. Having such connectivity will make time-series data analysis easier. It will give an explanatory model that reflects what is considered normal or abnormal behaviour by the tree moisture.

There are obvious differences between tree trunks and soil structure. Soil consists of a mixture of minerals, air, water, and organic material that contributes to the bio content of the earth. This inhomogeneous mixture and the variation between different soil types makes variations in water retention capabilities similar between different regions. Soil humidity hugely affects agriculture due to its importance in the plant and tree life cycle [1]. The soil’s physical structure, such as spaces between particles, soil type (sand, loam, peat, clay, etc.), and drainage capability, affect water retention [2]. Therefore, knowing the soil’s physical characteristics, such as soil temperature, moisture content, and salinity, helps farmers care appropriately for their plants and trees. It enables a better understanding of irrigation needs regarding water quantities and watering intervals.

Contrary to the soil, the wood structure is more homogeneous, with a less dynamic structure nature. The wood tissue and stems are rigid and less affected by the external environmental variations. This implies that the humidity variation inside the tree trunks needs more time to be noticed via sensors.

The paper is organized as follows: Section 2 presents the recent literature on capacitive sensors; Section 3 explains the relation between capacitance and permittivity in wood trunks; Section 4 demonstrates the mathematical modelling of moisture variations; the circuit design and simulation results are shown in Section 5.

The research’s novelty and contributions can be summarised as follows:

(1) Design of a low cost noninvasive capacitive sensor with LoRa communication interface.
(2) Detection of anomalies using statistical-based approaches.
(3) Characterisation of moisture–capacitance relation using Taylor series approaches for more accurate differentiation between different models.

## 2. Literature Review

Using sensors in precision agriculture has been widely investigated in the literature, mostly using sensors to measure soil moisture [3,4,5] or using Frequency Domain Reflectometry (FDR) or Time Domain Reflectometry (TDR) to detect variations in the moisture content of soil [6,7]. There are approaches in the literature in which capacitive fringing fields use PCBs to measure the dielectric materials’ variations in the soil [8,9].

There is a wide application for capacitive sensors, such as the measurement of displacement, pressure, density, and humidity, measuring small capacitance values in the range of pico-farads (0.1–30 pF) with a resolution of femto farads. However, there are complications in the design and cost needed to accurately measure such small capacitances due to the need for highly accurate and sophisticated instrumentations. In [10,11,12,13,14,15,16], different techniques of capacitive sensors are presented; each is designed for a specific range of capacitance values.

In [10], a test chip designed and fabricated to evaluate measurement methods for small capacitances is described. It consists of metal-oxide-semiconductor (MOS) capacitors, metal-insulator-metal (MIM) capacitors, and a series of systematically varying capacitance structures. The measured capacitance Devices Under Test (DUT) ranges from 0.3 fF to 1.2 pF. This test chip enables the measurement of variations in capacitance down to the 0.1 fF level.

Another method for measuring small capacitance values at high frequency without expensive test setups is phase measurement [11]. This method can measure various capacitances over a wide frequency range. In this process, the loss factor of the capacitance is also determined. This method’s theory of measuring capacitance by phase difference is based on the simple RC low-pass filter. A similar frequency measurement technique has been presented in [12]. Low-frequency and low-level capacitance variations are measured using a ‘lock-in detection” circuit with a feedback loop containing an integrator and a modulator to set the capacitance mean value to zero. This approach gives an excellent Signal to Noise Ratio (SNR) with high sensitivity. Capacitance variations can be in the range of 100 ppm of the mean value, while the frequency of the variations can be as low as 0.1 Hz. The results show that a small capacitance deviation of about 10 fF can be measured accurately.

In another design, a simple interface circuit to measure the minimal variation of capacitive sensors is proposed [13], which is suitable for industrial measurements. A higher resolution (step size) can be achieved using a computer-controlled 24-bit A/D converter. It also can be used with various types of capacitive sensors. The main advantage of this method is that the measurement obtained through this interface circuit is independent of the sensor’s initial capacitance value. Moreover, the double differential operating principle minimizes the error caused by coupling and stray capacitance in sensor probes to avoid frequent calibration. The results show that a yield of high-precision measurement can be achieved for floating capacitive sensors regardless of the initial sensor capacitance and type of construction. Furthermore, the front end of the interface circuit can be easily modified to measure minimal changes in inductive or resistive sensors.

It is important to note that different trees have different wood structures and water retention capabilities. Hence, the moisture content measurement for each tree type needs to be calibrated to better understand its physical meaning in terms of hydration. In [14,15], a calibration method for capacitive sensors designed for soil moisture measurements is presented. In [14], the development of a semi-automatic virtual instrumentation system using dual sensors with a confidence-weighted average to minimize the variation in sensor readings is documented. In [15], a calibration-free method for capacitive sensors was proposed. The authors derived a water-capacitance model related to specifics and content type, and other models for other sand or soil types. The model’s Root Mean Squared Error (RMSE) compared to the measured values was around 2%.

In [16], a technique to measure the variation in current with respect to humidity content was proposed. low current values (less than ten pA) can be accurately measured using a 20 pF air-gap capacitor. In such measurements, an unknown current I is applied to charge reference capacitor C, and the resulting linear voltage ramp dV/dt across the capacitor is measured with an accurate voltmeter. The current can then be determined via Equation (1). The coaxial Air-Gap capacitor is a feedback capacitor from a vibrating reed electrometer. During the normal operation of the electrometer, it is surrounded by a grounded metal shield to get a better-defined capacitance value. The capacitor plates are gold-plated to reduce the surface effects of contaminants with a nominal capacitance value of 20 pF.

I = C · dV/dt(1)

In another approach [17], capacitance to voltage converter design is introduced to measure the slight change in capacitance produced by human body movement. The main application of this work is to develop a reliable capacitance sensing system for measuring very small capacitive changes at low frequencies for human interface applications; the output voltage waveform can measure the capacitive variations. The simulation results show that a minimal capacitive variation, within the range of 10 pF to 100 pF, can be detected using the developed capacitive sensing method.

Despite having accurate measurements of sensory data to better decide irrigation needs, there is a need for a detection mechanism for abnormal sensor readings; the anomaly detection techniques for sensors, in general, vary according to the algorithm followed. Some follow the machine learning approaches, as in [18], or use a statistical anomaly detection method, as in [19]. All are designed to split the true positive from false positive sensor data.

The variations in time or frequency or phase to detect the moisture changes need to be identified as in [20,21] then data need to be mathematically modeled using tools as in [22,23] no matter whether it was capacitive or resistive sensor as in [24]. A summary of the literature in terms of the technology used, the medium of the sensors, and the cost of the solution is shown in Table 1.

## 3. Wood Dielectric and Permittivity

**Figure 2:**
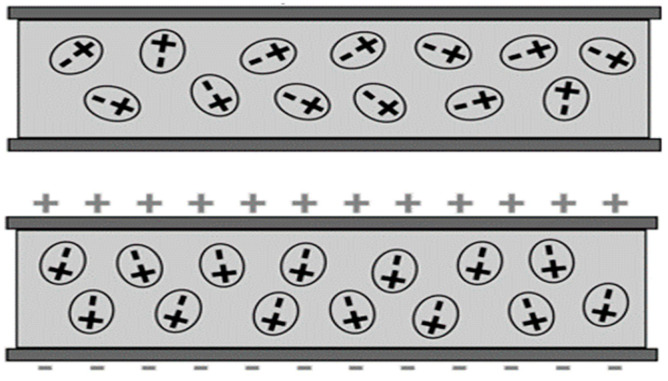
Dielectric material polarization.

Dielectric materials have low electrical conductivity, so no free electrons contribute to current flow. The exposure of dielectric material to an electric field causes dielectric polarisation; as shown in Figure 2, the induced external electromagnetic field polarises the dielectric material, and this induces an opposite reversely directed electromagnetic field. The polarisation helps in storing energy inside the material.

Permittivity is a measure of the electric polarisability of a dielectric material. When an electric field is applied to a material with high permittivity, it polarises more than a material with low permittivity. Permittivity can be used to determine the capacitance of two plates sandwiching the material. In electromagnetism, the electric displacement field represents the distribution of electric charges in each medium resulting from an externally induced electric field. This distribution includes charge migration and electric dipole reorientation, where the permittivity ε is a scalar [20].

Permittivity can vary with the medium’s position, the field’s frequency, humidity, and temperature. In a nonlinear medium, permittivity could also depend on the strength of the electric field. The water content in the trunk of a tree affects permittivity. This will appear as changes in the frequency measurements due to the polarisation mechanism [32]. An oscillator is used to produce a repetitive sinusoidal waveform; the frequency of oscillation decreases as the electrical permittivity increases. Water has a relative permittivity of 80, so the water content in a material affects its relative permittivity. The better the permittivity, the higher the capacitance, which leads to a slightly better dielectric. This allows the electric field to propagate easily from one side to another. Thus, the capacitance of the capacitor affects how fast the oscillation frequency changes.

Volumetric water content (VWC) is the ratio of the volume of water to the unit volume of soil. Volumetric water content can be expressed as a ratio, percentage, or water depth per soil depth (assuming a unit surface area). Capacitive humidity sensors consist of two electrodes separated by a dielectric. Humidity is measured by applying AC voltage between the electrodes and ascertaining the change in capacitance between the electrodes resulting from a change in permittivity due to moisture absorption. A built-in IC converts the capacitance values obtained via the sensor into a voltage signal whose frequency changes are the source of the humidity reads of the sensor. Frequency decreases as water content increases.

Compared to resistive sensors, capacitive sensors are a better option for measuring VWC due to anti-corrosion probes; they also produce analogue signals, and their metal parts are not exposed to water, which tends to operate the sensor for a long time. Table 2 shows sample permittivity measurements for different trees of the same type (i.e., *Melia azedarach*) that grow in a semi-arid land climate; some sensors are fitted over the same tree. The first columns represent the tree type and tree ID, and the second columns represent the fitted sensor ID—A3, for example, represent a single tree of type A and fitted with four sensors (3, 4, 5, 6) over its trunks and branches. We used a single tree type for this research, specifically *Melia azedarach*, due to its being widespread as forestry cover and to better detect anomalies, as shown later. Finally, the capacitance values are measured using a portable LCR meter over 100 kHz.

**Figure 3:**
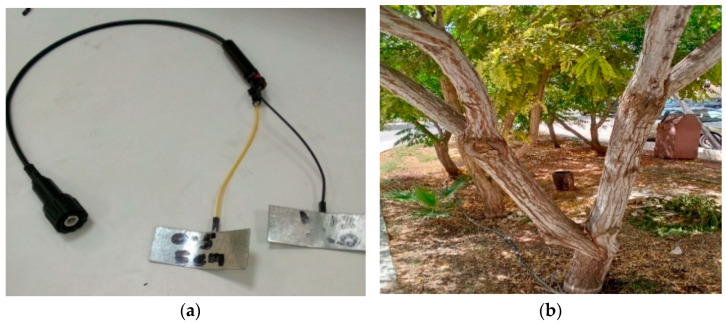
(**a**) Coaxial cable fitted to probes (**b**) Sample tree used in the experiment with multiple trunks.

The data were collected from five trees of three types, each fitted with single or multiple sensors (as indicated by the first three columns from the left of Table 2). Different probes were used for different plate sizes. To normalise the results, the plate size was calculated and the distance between plates measured using the trunk diameter. Figure 3a shows a sample tree with multiple branches, and Figure 3b shows the capacitive probe used, to neutralise the stray capacitance of the wires.

To have measurements that neutralise the environmental effect, such as ambient temperature and moisture, a lab-controlled experiment was developed. Small pieces of wood were taken from the same tree type, then weighed, dried, and weighed again. The dry-then-weigh process was repeated until there was no change in the weight, indicating that the wood was completely dry. This allowed the original moisture content to be calculated based on the (EN 13183-1:2002) standard.

The moisture content (MC) percentage was then calculated using Equation (2).

(2) $$MC\%=\frac{m_{1}-m_{0}}{m_{0}}*100$$

where *m*_1_ is the weight of the moist sample, and *m*_0_ is the weight of the dehydrated sample. The drying and weighing process is explained in detail in the EN 13183-1:2002 standard.

The *MC* estimation [20] can be used to determine the changes in the permittivity of the dielectric material by considering two frequencies (*f*_1_*, f*_2_), and can be written as shown in Equation (3).

(3) $$(\varepsilon_{r1}-\varepsilon_{r2})=\left(C_{1}-C_{2}\right)\frac{d}{\varepsilon_{0}A}$$

where *ε_r_*_1_ and *ε_r_*_2_ are the relative permittivity due to induced frequencies *f*_1_ and *f*_2_, *C*_1_ and *C*_2_ are the measured capacitances due to *f*_1_ and *f*_2_ consequently, *d* is the distance between probes, ε_0_ is the permittivity of the free air, and *A* is the probe cross-sectional area. Equation (3) can be transformed as a function of the first frequency, the dissipation factor and phase angle (*D*_1_, θ_1_), and the second frequency (*D*_2_, θ_2_), as shown in Equation (4).

(4) $$MC=A_{0}+A_{1}\left(C_{1}-C_{2}\right)+A_{2}{\left(C_{1}-C_{2}\right)}^{2}+A_{3}\left\{\left[\frac{\theta_{1}-\theta_{2}}{\left(C_{1}-C_{2}\right)+2\left(D_{1}-D_{2}\right)}\right]-\left(C_{1}-C_{2}\right)\left(D_{1}-D_{2}\right)\right\}$$

For single frequency, this can be further simplified to Equation (5).

(5) $$MC=A_{0}+A_{1}\left(C\right)+A_{2}{\left(C\right)}^{2}$$

Although this research was conducted over one single tree type (*Melia azedarach*), changes in the probe cross sectional area (due to using different probe sizes), changes in the distance between the two probes (due to a change in the trunk diameter), or changes in the permittivity (due to a change in the tree type and water content) will certainly change the capacitance reading, as indicated in relation (*C = ε A/d*), in which ε is the permittivity, *A* is the probe cross sectional area, *d* is the distance between the probes, and *C* is the measured capacitance. Therefore, knowing the area of the probes and the distance between them and then measuring the capacitance using an LCR meter will give an accurate estimate of the permittivity; this procedure neutralises the changes in trunk diameters. When it comes to moisture content calculation, Equation (5) gives a general form of the relation with undefined coefficients; we used an empirical approach to derive the coefficients using the Taylor series expansion method, which is valid for any tree type with any trunk diameter.

## 4. Mathematical Modeling of Moisture

Equation (5) provides the direct nonlinear relation combining the capacitance values with the moisture content percentage. Knowing that the probes give Pico Farad capacitance, as indicated in Table 2, means that there is a need to design a circuit that can measure a range of capacitances to reflect the capacitance variation (more on this in a later section). For now, we need to better link the coefficients of (5) to give an indication of the physical characteristics of the wood trunks.

**Figure 4:**
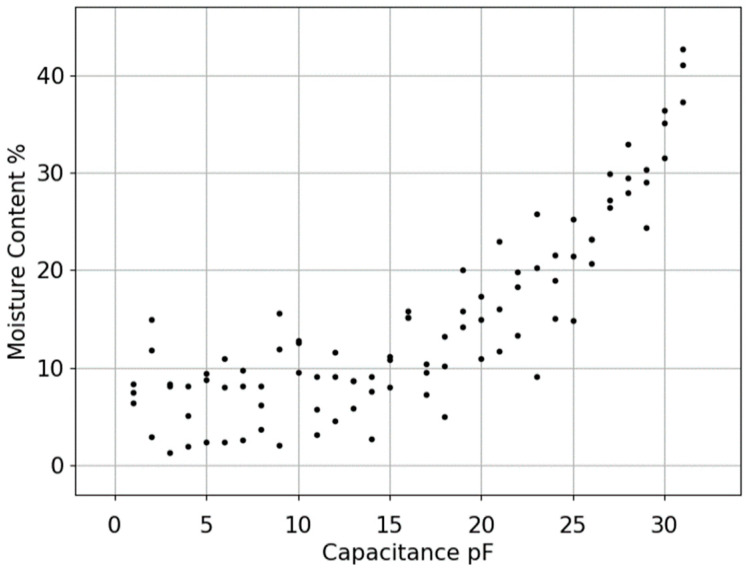
Scattered data measured to show relation between capacitance and moisture.

To model the behaviour of the moisture content change in the tree, multiple samples were collected and their moisture content measured using an LCR meter. Having both the moisture percentage and capacitance values helped to better visualise the relation, as shown in Figure 4.

The approach used in this paper for curve fitting is based on the error associated with the data points shown in Figure 4 (i.e., measured values). When the data have a significant error or noise, a single curve that represents the general trend of the data is derived. In this case the curve is designed in a way that follows the pattern of the points. This approach is known as least squares regression.

To interpolate the measured data, there is a need for a fitting algorithm to plot the curve on top of the scattered data (measured data). The best fit that reflects the data trend of the measured data is the exponential function that shows the relationship between capacitance and moisture. The general form of the exponential function is shown in Equation (6) [21]:

(6) $$y=\alpha e^{\beta x}$$

where *α* is the value of *y* when *x* is set to Zero and *β* is the growth factor during each unit period of time. To find the values of *α* and *β*, the above equation is linearised, as shown in Equation (7).

(7) lny=lnα+βx

After linearising the equation, we made the following substitutions to obtain Equation (11):

(8) Y=lny

(9) $$\ln\alpha =a_{0}\;\;\;$$

(10) $$\beta =a_{1}$$

(11) $$Y=a_{0}+a_{1}x_{i}+e_{i}$$

where *e_i_* denotes the error (difference between the measured (actual) and calculated values) as shown in Equation (12):

(12) $$e_{i}=y_{i,measured}-y_{i,model}=y_{i}-a_{0}-a_{1}x_{i}$$

let *S_r_* denote the sum of the squares of the residuals (errors) for *n* data points with the linear model, as shown in Equation (13)

(13) $$S_{r}=\sum_{i=1}^{n}e_{i}{}^{2}=\sum_{i=1}^{n}{\left(y_{i}-a_{0}-a_{1}x_{i}\right)}^{2}$$

To determine values for *a*_0_ and *a*_1_ in Equation (13) the derivative with respect to each coefficient was obtained as in Equations (14) and (15):

(14) $$\frac{\partial S_{r}}{\partial a_{0}}=-2\sum_{i=1}^{n}(y_{i}-a_{0}-a_{1}x_{i})$$

(15) $$\frac{\partial S_{r}}{\partial a_{1}}=-2x_{i}\sum_{i=1}^{n}(y_{i}-a_{0}-a_{1}x_{i})$$

In order to minimize *S_r_*, Equations (14) and (15) are set to zero, and rearranged as follows in Equation (16):

(16) $$\left[\begin{array}{cc} n & \sum x_{i}\\ \sum x_{i} & \sum x_{2}^{i} \end{array}\right]\left[\begin{array}{c} a_{0}\\ a_{1} \end{array}\right]=\left[\begin{array}{c} \sum y_{i}\\ \sum x_{i}y_{i} \end{array}\right]$$

After applying the solution of Equation (16) using the input, the scattered data, the values of *α* and *β* were found to be 1.32 and 0.106, respectively. Therefore, the exponential relation between capacitance and moisture is shown in Equation (17).

(17) $$MC=1.32{\;e}^{0.106\;C}$$

Equation (17) can be mathematically expanded using Taylor series expansion, which is used in numerical methods to approximate any smooth function as a polynomial by predicting the function value at one point in terms of the function value and its derivatives at another point [32]. Taylor series expansion is selected due to the polynomial approximation it provides to get a form close to the expected single frequency relation demonstrated in Equation (5). Moreover, Taylor series is a computationally less complex approximation due to the similarities between the exponential function and its n^th^ derivatives counterparts; this is demonstrated later in Equation (25).

When the function f and its first derivatives are continuous on an interval containing *x_i_* and *x_i_*_+1_, then the value of the function at *x*_*i*+1_ is given by Equation (18):

(18) $$f\left(x_{i+1}\right)=f\left(x_{i}\right)+f^{\prime \left(x_{i}\right)}h+\frac{f^{\prime \prime \left(x_{i}\right)}}{2!}h^{2}+\frac{f^{\left(3\right)}\left(x_{i}\right)}{3!}h^{3}+\ldots +\frac{f^{\left(n\right)}\left(x_{i}\right)}{n!}h^{n}+R_{n}$$

where the remainder *R_n_* is defined as in Equation (19).

(19) $$R_{n}=\frac{f^{\left(n+1\right)}\left(\xi \right)}{\left(n+1\right)!}h^{n+1}$$

*ξ* is a value of *x* that lies somewhere between *x_i_* and *x_i_*_+1_, and *h* is the step size (the distance between *x_i_* and *x_i+_*_1_), as shown in Equation (20):

(20) $$h=\left(x_{i+1}-x_{i}\right)$$

The exponential function is then approximated using Taylor series expansion via the following substitutions:

(21) $$x_{i}=0$$

(22) $$x_{i+1}=x$$

(23) h=x

Then, the function *f(x_i_*_+1_*)* becomes as in Equation (24).

(24) $$f\left(x_{i+1}\right)=f\left(x\right)=e^{x}$$

The initial value of the function and its derivative when *x_i_* is set to 0 becomes equal to 1 as shown in Equation (25).

(25) $$f\left(0\right)=f^{\prime }\left(0\right)=f^{\prime \prime }\left(0\right)=\ldots =f^{\left(n\right)}\left(0\right)=1$$

Following the substitution of the previous initial values and derivatives, *e^x^* can be expanded using the general form of complete Taylor series shown in Equation (18), as shown in Equation (26).

(26) $$e^{x}=1+\frac{x}{1!}+\frac{x^{2}}{2!}+\frac{x^{3}}{3!}+\ldots$$

In the current work the exponential function in Equation (17) was expanded using a second-order Taylor series expansion, as shown in Equation (27).

(27) $$MC=1.32+0.13992\;C+0.0074\;C^{2}$$

Comparing Equation (27) with Equation (5) when both are considered equivalent, we can define the coefficients {*A*0, *A*1, *A*2} of Equation (5) as the Tree Trunk Moisture Coefficient Vector (*TTMCV*) to be as defined in Equation (28).

(28) TTMCV={A0,A1,A2}={1.32,0.1399,0.0074}

**Figure 5:**
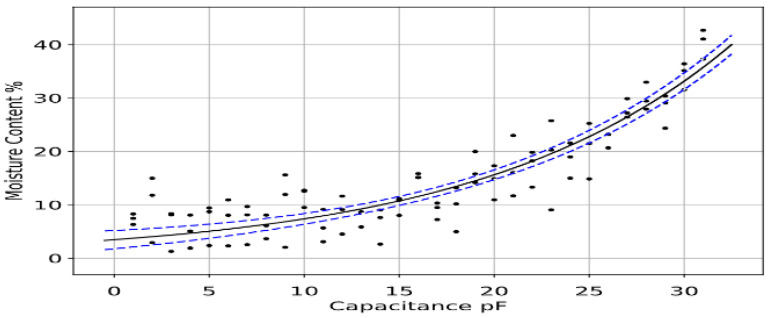
Fitted exponential curve over the experimental data.

The fitted curve over the collected data within a 95% confidence interval is shown in Figure 5, the blue dotted lines represent eh confidence interval boundaries. The statistical results associated with this fitted curve will be used as an example for other fitted curves as it represents all collected data for five trees of the same type (i.e., *Melia azedarach*).

Since the TTMCV is a unique characteristic for each tree as it tells the different abilities of each tree in terms of water retention compared to different tree species, it can also be used to detect anomalies once the normal expected behaviour is well established. For this purpose, we fitted the sensor into multiple healthy trees of the same type and collected the sensor data to visualise the data to show the relation between capacitance and moisture content; then, the data was averaged to remove unwanted deviations and to reflect the normal behaviour of expected moisture in the same tree type planted within the same geographical area under the same weather and soil conditions. TTMC was then generated following determining the coefficients of the fitted exponential curve.

**Figure 6:**
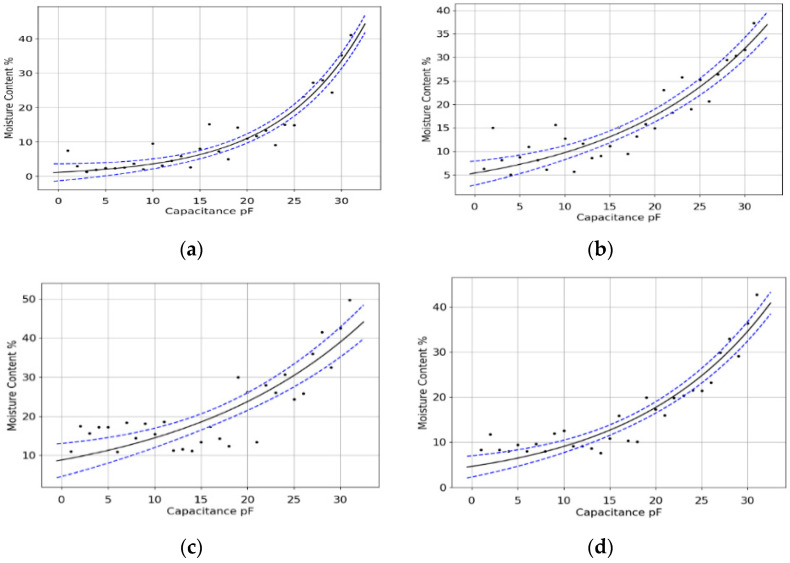
(**a**) infected tree data (**b**) healthy tree data (**c**) data for healthy tree with poor probe insulation (**d**) averaged healthy trees data.

When it comes to anomalies due to trunk physical deformations or diseases, it is better to have separate fitted curves for each case and derive the TTMCV value for each one of them, then compare it statistically to healthy TTMCV values in the database. We fitted one sensor to a tree trunk with major physical deformation, another sensor to a normal healthy trunk, and the third to a similar healthy trunk with moisture accumulated around the probes. After splitting the measured data for each sensor and fitting the exponential curve over it we obtained the results shown in Figure 6a–c; similarly, the average of the entire collected data from all healthy trees is shown in Figure 6d, the confidence interval boundary lines are shown in blue dashed format.

Table 3 shows the statistics for each fitted curve and the associated TTMC vector for each one of them. The results show that there is a clear distinction between each tree, which is reflected in many parameters: the most important parameters are the coefficient values (*α*, *β*) of the fitted curve in addition to the coefficient covariance matrix, which shows all the variances of coefficients within the selected confidence interval. The matrix gives the values of variance of each coefficient with itself (variance of *α* and variance of *β* on the diagonal) and the variance of one coefficient with respect to the other (referred as covariance values). The covariance matrix gives an indication of how widely the coefficients are deviated from the mean and hence gives a good enough statistical-based approach to compare the similarity or deviation of any two datasets.

The collected datasets denoted by DS in Table 3 represent samples of data measured using the developed sensor: DS4 represents the averaged collected data from healthy trees of the same type, while the remaining datasets belong to one healthy tree (represented by DS2) and two defected ones due to deformations in the trunk or insect disease or accumulated moisture around the probes due to erosion or poor insulation (represented by DS1 and DS3); all samples are for the same type of trees. Using statistical-based approaches helps in deciding which of the three datasets (DS1–DS3) is similar to DS4 and whether it is classified as healthy or not.

We cannot rely on Chi-squared, R-squared, or RMSE values in Table 3 to check the similarities between any two curves, as they reflect the accuracy of the fitting rather than the fitting curve coefficients; in other words, we may have two different fitted curves with same R-squared or RMSE values. Similarly, we cannot check the similarity or differences between two datasets using capacitance variance and standard deviations or the moisture content variance and standard deviations, as such values are either equal or close to each other for all datasets, for example, during the capacitance measuring procedure; fixed steps of capacitances were selected during the drying process, which made the variance of capacitance almost the same for all datasets. This means that we need to have a statistical approach that reflects the fitted curve coefficients and the variations in such coefficients at the same time. For this purpose, we use the coefficient covariance matrix elements for each dataset. For a fair comparison, we find for each dataset the eigenvalues (as a reflection of the spreading magnitude for each coefficient) and eigenvectors (as a reflection of maximum spreading direction for each coefficient) along with other statistics derived from the coefficient covariance matrix, as shown in Table 4.

The sensor readings due to improper sensor calibration, malfunctioning, or probe misalignment, poor insulation are considered possible anomalies. Therefore, DS3 is defined as a dataset in which the sensor probes are not well insulated which may cause humidity and mist to accumulate on the probe surface. The detection of anomalies needs a full dataset to conduct a statistical-based approach, which means that the sensor data need to be collected first until we have enough to extract the statistical features. We cannot, in real time, apply the detection using instant sensor reading. However, machine learning approaches can be followed to have near real time detection over certain time window sizes.

For comparison purposes, Table 5 shows the absolute difference between datasets parameters, mainly the trace (sum of λ_1_ and λ_2_) and eigenvectors *v*_1_ and *v*_2_; differences have been calculated between each data set and DS4. The results clearly show (shaded in Table 5) that DS2 is the closest statistically to DS4, which complies with the fact that both datasets belong to the data collected from healthy trees. Similarly, the deviations (especially in the eigenvector value of the first coefficient) detect the anomalies easily.

## 5. Circuit Design and Simulation Results

**Figure 7:**
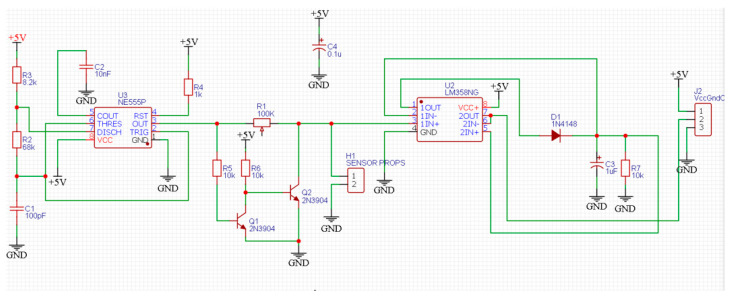
Schematic diagram of the sensor circuit.

This section presents a simulation of the proposed circuit using MULTISIM^®^ to measure the capacitance value, as shown in Figure 7. The design relies on 555 timer circuit that is calibrated to generate 100 kHz frequency square signal. It is directly applied through a potentiometer, for calibration purposes, to the probes that are supposed to be fitted into the tree trunk. Then, a low pass filtration and two-stage amplification are imposed to increase the variation between the capacitance and output DC voltage step.

**Figure 8:**
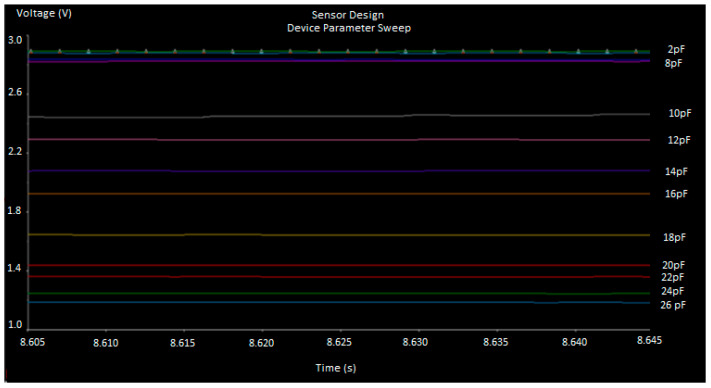
Simulation results showing the variation in sensor output voltage while changing the capacitance value.

Following the final amplification stage, shown in Figure 7, it becomes clear that the distinction between capacitance and output voltage is better deviated, as shown in Figure 8. The horizontal lines represent the output DC voltage with respect to the capacitance value. The simulation has swept the capacitance values between 2 pF and 26 pF in steps of 2 pFs, with the top-line representing the lowest capacitance (2 pF) and the bottom one being the highest one (26 pF). It is noted that the voltage difference between small capacitances (from 2 pF to 8 pF) is minimal, whereas it is more noticeable for higher ranges (from 10 pF to 26 pF). The dynamic range of the sensor output DC voltage is important and gives an indication of which capacitances are more feasible to be measured. Finally, the Bill of Material (BOM) is shown in Table 6, which shows the Sensor board electronics costs only (not counting the casing, microcontroller, LoRa interface, PCB printing board cost).

**Figure 9:**
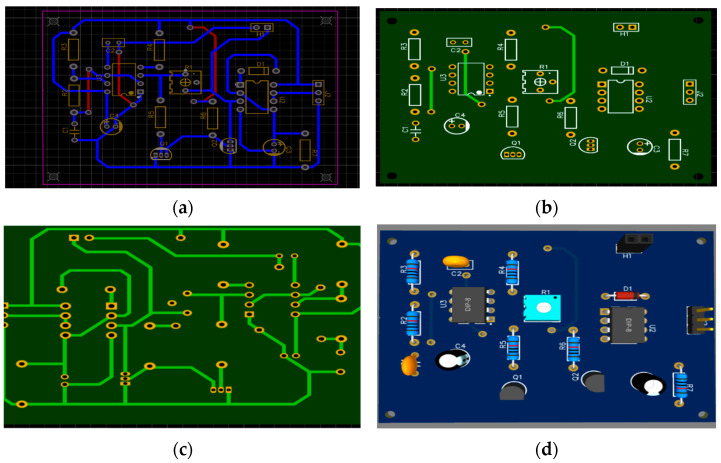
The PCB design (**a**) two layers (**b**) top layer (**c**) bottom layer (**d**) 3D design.

**Figure 10:**
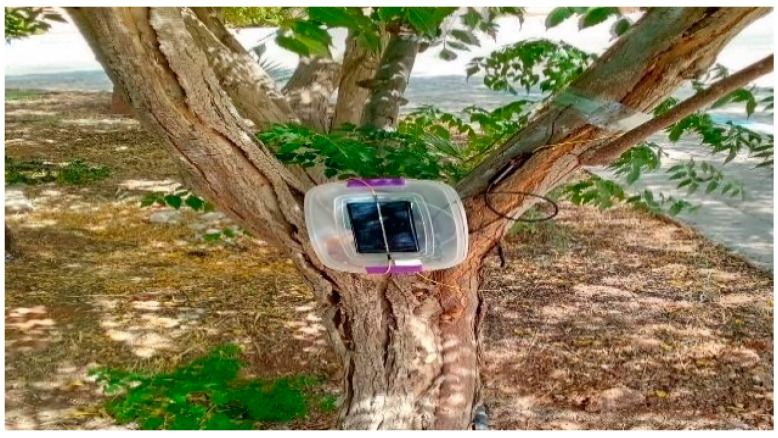
Real prototype fitted on a tree.

The PCB design was conducted using EasyEDA, the double layer PCB design. The top layer, bottom layer, and 3D design are all shown in Figure 9, using FR4 boards. Finally, the fitted sensor prototype, along with probes and a LoRa communication interface and antenna, is shown in Figure 10. A solar panel with an ESP32 microcontroller and a lithium battery with a LoRa communication interface (868 MHz antennae) has been fitted inside a waterproof container and data are transmitted successfully to the LoRa gateway, located 600 m away, The sensor output is connected to LoRa transmitter using Heltec Cubcell, which is an ESP32-based development board that is equipped with LoRa transceiver, in which we have connected the sensor to the Analogue to Digital Converter (ADC) pin; the code written is set to send the data periodically into the LoRa gateway that is interfaced with a Raspberry Pi board for data storage. The maximum distance that has been achieved between sensor and gateway is 700 m; beyond this, data retransmission occurs frequently due to the low SNR levels of the signals. As a matter of fact, the LoRa standard uses Chirp Spread Spectrum (CSS) modulation, which uses wideband spectrum to make it resilient to multipath and Doppler effects but with low data rates; this makes it the optimum choice for sending sensor data over long range. LoRa allows multiple Spreading Factor (SF) configurations that need to be picked for optimum distance and required data rate; for this experiment scenario, the distance achieved could be better enhanced via a higher gain antenna.

## 6. Conclusions

The moisture of tree trunks is a better estimate of tree hydration compared to simply measuring the soil moisture content. This paper describes a methodology to determine the moisture content inside tree trunks via quantifying the relation between humidity, permittivity, capacitance, and output voltage. Using an empirical approach, the moisture and capacitance of multiple wood samples have been collected and a relation has been extracted; the mathematical model is highly correlated with the measured value considered a reliable approach. A new terminology has been defined (Tree Trunk Moisture Coefficient Vector, TTMCV) that reflects the physical characteristics of tree trunks, according to the capacitance and moisture content data, while a dedicated circuit has been designed to measure a wide scope of capacitance variation and the simulation results have shown that the proposed circuit can successfully distinguish between different moisture contents with a low RMSE value (around 0.02), compared to other approaches in the literature; despite being a low-cost sensor, the built prototype performed the experiment successfully. Finally, detailed statistical analysis that relies on Taylor series expansion of the fitted exponential curve proved that there is a clear distinction line that can split between the different datasets reflecting healthy trees, defected sensor, or infected trees. Such distinction is achieved via thorough analysis of eigenvectors and eigenvalues generated out of the covariance matrix of the fitting coefficients. Although the results showed that it is possible to measure the low capacitances of the selected tree type, there is a need to well calibrate the sensor to operate within the dynamic capacitance range if other tree types need to be taken into account. Another limitation that we encountered is that the sensor probes need to be well isolated to avoid the accumulation of moisture over their surface, which indicates anomalous results. In the future, there is a chance to improve this research via commercialising the prototype and conducting additional electromagnetic interference tests and durability tests.

## Figures and Tables

**Table 1:** Literature comparison.

Reference	Technology	Probes	Frequency	RMSE	Medium	Cost
[1]	Capacitive sensor	Annular Electrode	150 MHz	0.0423	soil	Medium
[3]	Coil induction coupling	coils	93 KHz	N.A	Soil	Low
[6]	FDR	Open ended coax	500 MHz	N.A	Soil	High
[7]	TDR	Open ended probe	100 MHz	N.A	Grass/Snow	Very high
[8]	Capacitive sensor	PCB	Low frequency	N.A	Soil/vegetation	Medium
[16]	Capacitive sensor	plates	1 KHz	N.A	Air Gap	Low
[25]	Capacitive sensor	plates	250 KHz	0.02–0.05	Soil	Low
[26]	Coil induction coupling	coils	500 KHz	0.021	Soil	Low
[27]	TDR	Open ended probe	70MHz-4 GHz	N.A	Tree	Very High
[28]	Remote Sensing	LIDAR	N.A	N.A	Tree	Very High
[29]	UHF RFID	RFID Tags	500 MHz	0.055	Tree	Very High
[30]	dispersion-based electromagnetic	Patch Antenna	4–5 GHz	N.A	Soil/Tree	Very High
[31]	dispersion-based electromagnetic (WiFi)	Patch Antenna	2.4 GHz	N.A	Tree	Very High
This Work	Capacitive sensor	plates	100 KHz	0.02	Tree	Very low

**Table 2:** Sample measured data.

Tree-Type-Sensor ID		Capacitance (PF)	Probe Size (cm^2^)	Trunk Diameter (cm)	Permittivity
A1	1	17.5	26	11.45	87
A2	2	683	8	7	67
A3	3	6.6	26	11.14	32
A3	4	9	10	7.63	77
A3	5	4.4	8	7.63	47
A3	6	3.9	8	7.63	42
A4	7	22.5	26	11.45	111
A5	8	9.5	26	10.82	44

**Table 3:** Collected datasets statistics.

Tree-Type- Sensor ID	DS1	DS2	DS3	DSavg
Chi-squared:	155.945	476.961	1013.777	266.366
R-squared:	0.949	0.846	0.723	0.891
Root Mean Squared Error (RMSE):	2.242	3.922	5.718	2.979
α =	1.697	4.974	7.954	4.622
β =	9.897 × 10^−2^	6.598 × 10^−2^	5.413 × 10^−2^	6.843 × 10^−2^
Coefficient Covariance Matrix	$\left[\begin{array}{cc} 0.012\; & -0.0002\\ -2.60\times 10^{-4}\; & 5.78\times 10^{-6} \end{array}\right]$	$\left[\begin{array}{cc} 0.032 & -0.0002\\ -2.53\times 10^{-4}\; & 2.08\times 10^{-6} \end{array}\right]$	$\left[\begin{array}{cc} 0.044 & -22\times 10^{-5}\\ -2.21\times 10^{-4}\; & 1.19\times 10^{-6} \end{array}\right]$	$\left[\begin{array}{cc} 0.033 & -289\times 10^{-6}\\ -2.89\times 10^{-4} & 2.64\times 10^{-6} \end{array}\right]$
Capacitance Variance	80	80	80	74.91
Moisture Variance	99.54999	99.89192	117.9799	81.21409
Capacitance standard deviation	8.944272	8.944272	8.944272	8.655441
Moisture standard deviation	9.977474	9.994594	10.86185	9.011886
TTMCV vector	$\begin{array}{ccc} {}[1.697 & 0.1679 & 0.0083] \end{array}$	$\begin{array}{ccc} {}[4.974 & 0.328 & 0.0108] \end{array}$	$\begin{array}{ccc} {}[7.954 & 0.4305 & 0.0116] \end{array}$	$\begin{array}{ccc} {}[4.622 & 0.3162 & 0.0108] \end{array}$

**Table 4:** Covariance matrix statistics.

	DS1	DS2	DS3	DS4
Trace	0.0120058	0.0320021	0.0440012	0.0330026
Determinant	1.736 × 10^−8^	1.596 × 10^−8^	3.74 × 10^−9^	3.599 × 10^−9^
Characteristic Polynomial	λ^2^ − 0.0120058λ + 1.736 × 10^−8^	λ^2^ − 0.0320021λ + 1.596 × 10^−8^	λ^2^—0.0440012 λ + 3.74 × 10^−9^	λ2—0.0330026 λ + 3.599 × 10^−9^
First eigenvalue	λ_1_ ≈ 0.0120043	λ_1_ ≈ 0.0320016	λ_1_ ≈ 0.0440011	λ_1_ ≈ 0.0330025
Second eigenvalue	λ_2_ ≈ 0.144614 × 10^−8^	λ_2_ ≈ 4.98725 × 10^−7^	λ_2_ ≈ 84.9979 × 10^−7^	λ_2_ ≈ 1.09052 × 10^−7^
First eigen vector	*v*_1_ ≈ (−46.1483, 1)	*v*_1_ ≈ (−126.48, 1)	*v*_1_ ≈ (−199.095, 1)	*v*_1_ ≈ (−114.186, 1)
Second eigenvector	*v*_2_ ≈ (0.0166687, 1)	*v*_2_ ≈ (0.0062501, 1)	*v*_2_ ≈ (0.00500001, 1)	*v*_2_ ≈ (0.0087576, 1)

**Table 5:** Absolute difference between dataset parameters.

		DS1			DS2			DS3		
		Trace	*v*_1_	*v*_2_	Trace	*v*_1_	*v*_2_	Trace	*v*_1_	*v*_2_
DS4	Trace	0.0209	n.a	n.a	0.001	n.a	n.a	0.0109	n.a	n.a
	*v*_1_	n.a	68.04	n.a	n.a	12.29	n.a	n.a	85.09	n.a
	*v*_2_	n.a	n.a	0.00791	n.a	n.a	0.0025	n.a	n.a	0.00375

**Table 6:** Bill of material for the sensor board components.

ID	Name	Designator	Quantity	Manufacturer Part	Price(USD)
1	100 pF	C1	1	CC1H101KC74DSL4B10MN	0.02
2	10 nF	C2	1	CC1H103ZA1FD3F4D10MF	0.02
3	1 uF	C3	1	KF010M050C110A	0.06
4	0.1 u	C4	1	0.1UF 50V	0.02
5	1N4148	D1	1	1N4148	0.01
6	SENSOR PROPS	H1	1	N.A	0.06
7	VccGndOut	J2	1	N.A	0.31
8	2N3904	Q1, Q2	2	2N3904	0.03
9	100 K	R1	1	3362P-1-104LF	0.46
10	68 k	R2	1	MF1/4W-20Ω ± 1%T52	0.01
11	8.2 k	R3	1	MF1/4W-20Ω ± 1%T52	0.01
12	1 k	R4	1	MF1/4W-20Ω ± 1%T52	0.01
13	10 k	R5, R6, R7	3	MF1/4W-20Ω ± 1%T52	0.01
14	LM358NG	U2	1	LM358NG	0.5
15	NE555P	U3	1	NE555P	0.48
TOTAL				2.01USD	
